# Assessment of normal hemodynamic profile of mechanical pulmonary prosthesis by doppler echocardiography: a prospective cross-sectional study

**DOI:** 10.1186/s12947-020-00196-0

**Published:** 2020-05-15

**Authors:** Maryam Shojaeifard, Ali Daryanavard, Arman Karimi Behnagh, Maryam Moradian, Sajjad Erami, Hossein Dehghani Mohammad Abadi

**Affiliations:** 1 Echocardiography Research Center, Rajaie Cardiovascular, Medical, and Research Center, Iran University of Medical Sciences, Tehran, Iran; 2 Rajaie Cardiovascular, Medical, and Research center, Iran university of medical sciences, Tehran, Iran; 3 Shahid Sadoughi University of Medical Sciences, Yazd, Iran; 4 Shahid Sadoughi Hospital, Shahid Sadoughi University of Medical Sciences, Yazd, Iran

**Keywords:** Pulmonary mechanical prosthesis, Doppler-echocardiographic, Congenital heart diseases, Normal hemodynamic profile

## Abstract

**Objectives:**

Very few reports have described the Doppler-derived echocardiographic parameters for mechanical pulmonary valve prosthesis (MPVP). This study aims to describe the normal Doppler hemodynamic profile of MPVP using Doppler echocardiography.

**Methods:**

The current prospective, single center observational study enrolled 108 patients who underwent pulmonary valve replacement (PVR) surgery for the first time and had a normally functioning prosthesis post-operation. The hemodynamic performance of MPVPs, considering flow dependent and flow independent parameters, was evaluated at two follow-up points, at week one and week four post-operation. All assessments were conducted by an experienced echocardiographer.

**Results:**

The mean age (±SD) of the participants was 26.4 (±8.98). Tetralogy of Fallot (ToF) was the most common underlying disease leading to PVR, with a prevalence of 88%. At first week post-operation, measurement of indices reported the following values (±SD): peak pressure gradient (PPG): 18.51(±7.64) mm Hg; mean pressure gradient (MPG): 10.88(±5.62) mm Hg; peak velocity (PV): 1.97(±0.43)m/s; doppler velocity index (DVI): 0.61(±18); pulmonary velocity acceleration time (PVAT): 87.35(±15.16) ms; effective orifice area (EOA): 2.98(±1.02) cm^2^;and effective orifice area to body surface area ratio (EOA/ BSA): 1.81(±0.62) cm^2^/m^2^. Comparing these measurements with those obtained from the second follow-up (at week four post-op) failed to hold significant difference in all values except for PVAT, which had increased from its primary value (*p* = 0.038). Also, right ventricular (RV) function showed significant improvement throughout the follow up period.

**Conclusion:**

The findings of this study help strengthen the previously scarce data pool and better establish the normal values for Doppler hemodynamics in mechanical pulmonary prosthesis.

## Introduction

In recent years, mortality rates due to congenital cardiac anomalies have significantly declined, leading to the prolongation of lifespan in affected individuals. Amongst such anomalies, congenital defects of the right-side chambers are commonly managed with Pulmonary Valve Replacement (PVR). Individuals with Tetralogy of Fallot (ToF), the most common and perhaps the most widely known cause of cyanotic congenital cardiac diseases, now enjoy a longer life as a result of recent advances [1]. However, no gift comes without a price and future complications commonly haunt the patients later on in life, rendering repeated PVR operations a rational go-to solution in such instances [2]. Re-operation, most commonly PVR, is performed on close to 50% of individuals with TOF [3].

Two main type of valvular replacements are available: Biologic and Mechanical. Although mechanical valves are more durable, the use of these prosthesis is associated with an increased risk for thrombotic events requiring lifelong anticoagulation therapy. This renders them less favourable over the alternate option. However, it has been suggested that the implication of proper anticoagulation therapies can significantly reduce the risk of thrombotic events [4].

The relatively less frequent application of mechanical valves has resulted in a lack of knowledge regarding the function of prosthetic valve replacements. Doppler echocardiography is the method of choice for evaluation, in both mechanical and biologic prosthesis, capable of assessing a wide range of haemodynamic parameters [5]. Similar to native valves, normal Doppler echocardiography profile can be measured for prosthetic transplants, however the achieved profile may be heavily influenced by a number of variables such as valve type, location, size as well as patient-specific factors [6].

Normal haemodynamic values have been previously determined for mitral and aortic valve prostheses [7]. However, the limited knowledge on mechanical pulmonary valve prostheses (MPVP) has made the task of establishing normal haemodynamic values difficult in this type of prosthesis. To address this problem, we aimed to determine the haemodynamic profile of MPVPs in patients with desirable post-operation outcome, as established by clinical and echocardiographic assessments. In addition, a two-step evaluation was performed to negate the short-term effects of open-heart surgery on the haemodynamic profile of the prostheses, specifically on right ventricular function.

## Materials and method

This prospective cross-sectional study enrolled patients who underwent pulmonary valve replacement surgery at Shahid Rajaie Cardiovascular Medical and Research center, between August 2016 and September 2019. Data collection was performed at baseline (prior to surgery), at one week, time of discharge, and four weeks after surgery by trained staff with the use of a data collection checklist. This study was approved by the Ethics Committee of the Iran University of Medical Sciences.

### Study participants and implanted valve characteristics

Patients who underwent PVR operation for the first time and those with normally functioning mechanical valve in pulmonary position were eligible for this study. The exclusion criteria were Suboptimal postoperative imaging view and previous history of the Rastelli procedure or other valve prostheses. All of the valves assessed in this study were mechanical and obtained from three different manufacturers: St. Jude (St. Jude Medical, USA), Carbomedics (SORIN group, Italy) and On-X (On-X life technologies, USA), with a diameter size varying between 21 mm to 31 mm.

### Doppler echocardiographic procedure

Echocardiographic evaluation was performed in the left lateral decubitus position. In order to minimize anticipation bias, a single specialist was assigned with the assessment. In this study, we used a General Electric Vivid 7 phased array system (GE medical systems) equipped with a multi-frequency 2.5–3.4 MHz transducer. Apical, parasternal, long and short axis views were assessed via electrocardiogram signals of desirable quality and a frame rate of 45–55 FPS.

**Fig. 1:**
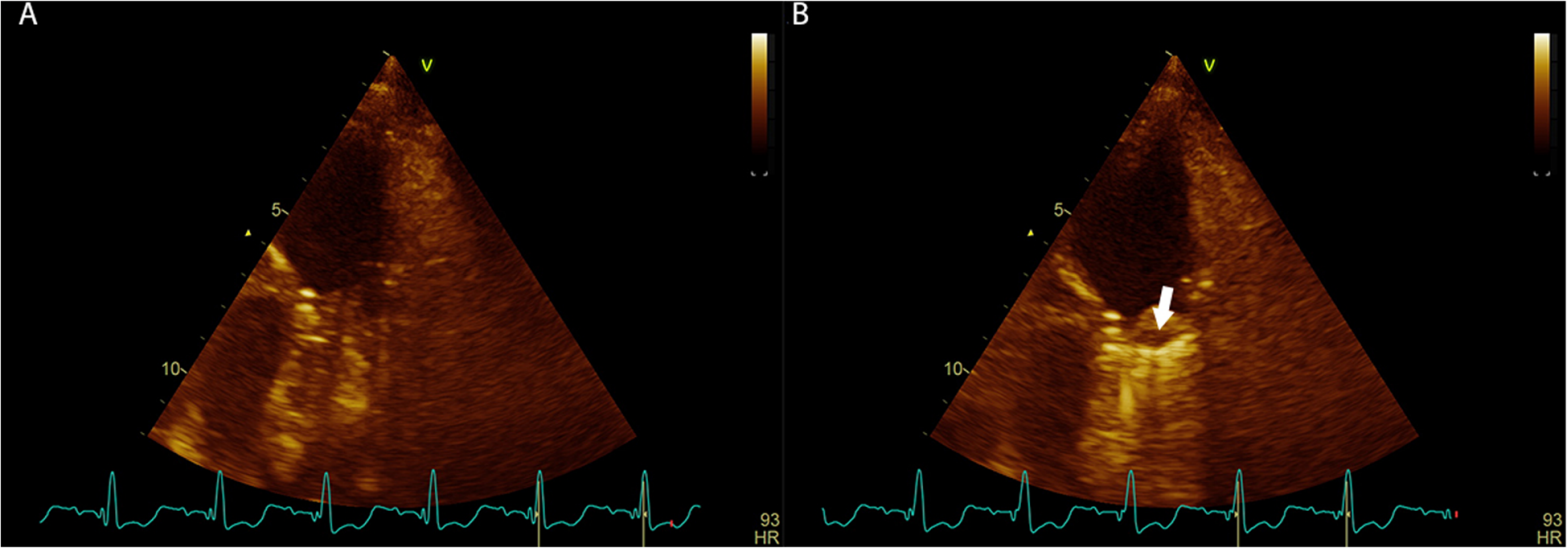
Normal functioning of mechanical prosthesis in pulmonary position. **a** Normal opening of the prosthesis. **b** Normal closing of the prosthesis

**Fig. 2:**
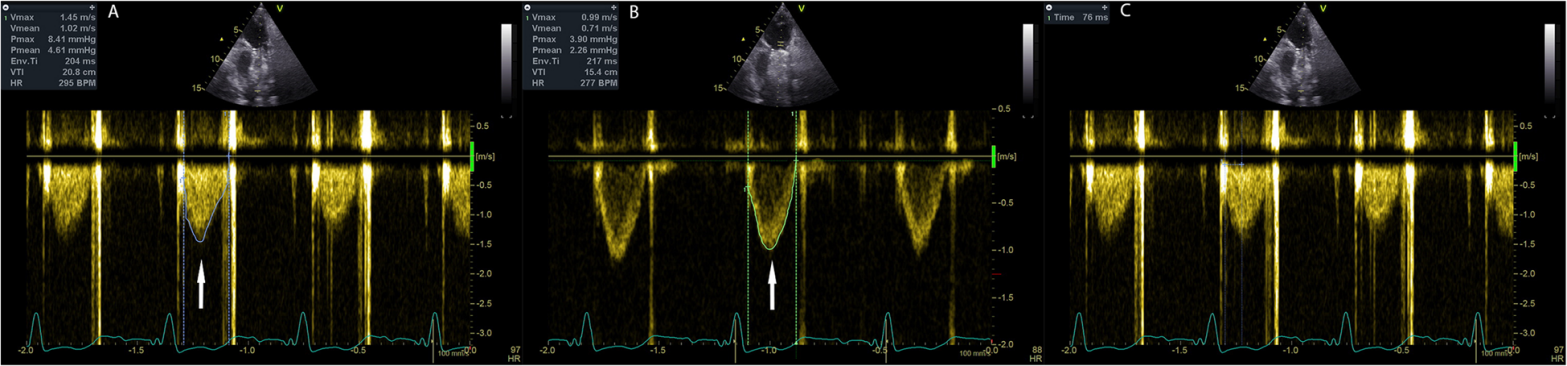
Doppler evaluation of a prosthetic pulmonary valve. **a** Tracing of pulmonary prosthesis doppler illustrating mean pressure gradient (MPG), peak pressure gradient (PPG) and peak velocity (PV) of prosthesis. **b** Calculation of Doppler velocity index by dividing right outflow tract (RVOT) VTI to pulmonary valve VTI **c** Obtaining acceleration time of mechanical prosthesis

Left ventricular Ejection Fraction was assessed optically using the Simpson method. Right ventricular (RV) function was determined via visual assessment, tricuspid annulus tissue systolic velocity and tricuspid annular plain systolic excursion. The Likert scale was used to present variables such as RV function, tricuspid regurgitation. Left ventricular and RV size and function were graded according to the recommendations of the American society of echocardiography. MPVPs were considered functional when the normal bi-leaflet motion of the prosthetic valve was observed in parasternal short axis or parasternal RV outflow view (see Fig. 1). Doppler evaluation of the prostheses assessed the flow velocity through the prosthesis via pulse waves, continuous Doppler and color Doppler imaging. In order to minimize angulation between Doppler beams and flow direction, multiple windows were used to obtain measurements and the highest overall velocity was recorded. Thereafter, Peak pressure gradient (PPG), Mean pressure gradient (MPG) and the outer edge of the peak velocity (PV) profile were drawn and applied for the mean and peak gradients, which were automatically calculated by computer software using the simplified Bernoulli equation, peak PVP velocity, Doppler velocity index (DVI) that means ration of right ventricle outflow tract (RVOT) velocity time integral (VTI),which measured by pulse wave Doppler, to PV VTI (by continuous Doppler), pulmonary velocity acceleration time (PVAT) that was obtained with time from the onset of flow to maximal velocity (see Fig. 2). Tricuspid regurgitation was determined using the Bernoulli equation. After obtaining these measurements, the EOA of the prostheses was determined using the continuity equation: cross-sectional area of RVOT*VTI of RVOT/VTI of prosthetic PV. The cross-sectional area of RVOT was calculated based on the size of the mechanical prosthetic.

### Statistical analysis

Continuous variables were reported as means or median, standard deviation (SD) or in ranges. Categorical and nominal data were reported with absolute and relative frequencies, and comparisons were carried out using chi-square test and the Fisher’s exact test. Furthermore, continues variables were compared using the paired t-Test or, in instances which the data failed to fulfil the parametric features, the Wilcoxon rank-sum test. Also, one-way analysis of variance followed by Tukey Post hoc test was used to compare parameters between valve types. The Kruskal-Wallis H test was considered when the data failed to fulfil parametric traits. The association between continues variables was assessed using the Pearson Correlation test. The Spearman rank-correlation test was used to compare nonparametric data or ordinal measurements. To show the 95% confidence interval for non-parametric data Bootstrap method at 1000 repetitions was implemented. Two-tailed analysis was performed in all instances and significance was determined at *P* < 0.05. Statistical analyses were carried out using SPSS software package v.20 for Windows (SPSS Inc., Chicago, Illinois).

## Results

**Fig. 3:**
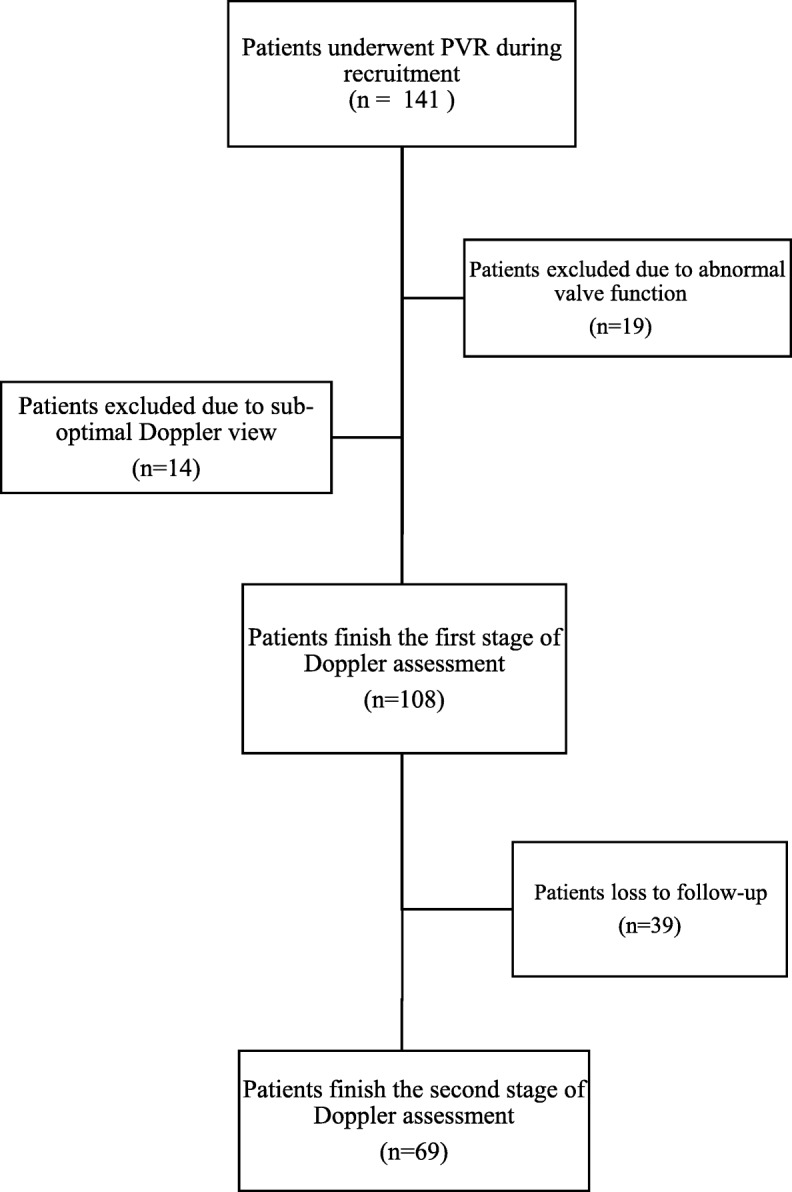
Study population selection flowchart, PVR: pulmonary valve replacement

During the recruitment period a total of 141 patients underwent PVR surgery. Of these cases 108 patients fulfill the eligibility criteria of this study and 33 patients were excluded. However, of all these 33 patients, non had history of previous PVR or any other cardiac surgery. Although all eligible cases were present for their first follow up, only 69 individuals visited the center for their second follow up. Lack of access to the remaining participants was the main cause of dropout (Fig. 3). Of the 108 participants, 69(63.9%) were male. The mean age(±SD) of the participants was 26.4(±8.98). At the time of echocardiographic evaluation, the global average heart rate was 80.25(±12.13) and a sinus rhythm was observed in all cases. In addition, all had participants exhibited normal LV diastolic function. Ejection fraction was measured in both stages. The mean left ventricular ejection fraction before and in first follow-up were 45.83% (±6.21) and 45.41% (± 5.96), respectively. ToF (*n* = 95;88%) was the most common underlying condition leading to PVR. Regarding the manufacturer of the prostheses, St Jude was implanted for 72 (66.7%) patients, which was the most frequent prosthesis used in this study. The baseline characteristics of the participants are summarized in Table 1.

**Table 1:** Baseline characteristics of participants

Age	26.41(±8.98)
Sex	
Male	69 (63.9%)
female	39 (36.1%)
Heart Rate	80.25(±12.13)
PVR indication	
Pulmonary insufficiency	105 (97.2%)
pulmonary stenosis	2 (1.9%)
Pulmonary insufficiency and stenosis	1 (0.9%)
Valve type	
SJ	73 (67.6%)
Carbomedics	19 (17.6%)
On-x	16 (14.8%)
Valve size	
21 mm	2 (1.9%)
23 mm	23 (21.3%)
25 mm	67 (62.0%)
27 mm	12 (11.1%)
29 mm	3 (2.8%)
31 mm	1 (0.9%)
Underlying Disease	
ToF	95 (88%)
pulmonary stenosis	8 (7.4%)
pulmonary insufficiency	3 (2.8%)
Atrioventricular septal defect	1 (0.9%)
Double outlet right ventricle	1 (0.9%)

Values are represented as mean(±SD) or n(%)

*PVR* pulmonary valve replacement, *SJ* St. Jude, *ToF* tetralogy of Fallot

The data on doppler measurement of all MPVPs for both stages was summarized in Table 2. At one-week post-operation, right ventricular size was 4.11(±0.77). Right ventricular function was also assessed at this follow-up point: mild in one patient (0.9%), mild to moderate in six patients (5.6%), moderate in 50 patients (46.3%), moderate to severe in 39 patients (36%) and severe in 12(11.1%) patients. The spearman correlation test between the valve sizes and hemodynamic indices revealed that valve size was positively correlated with EOA/BSA (r: 0.306; *P* = 0.001). In addition, a marginal correlation was observed between <PPG and valve size (r = − 0.175, *P* = 0.071). Moreover, the correlation between the right ventricular systolic pressure and the PPG showed a significant association of these two parameters (r: 0.350; P = 0.001). The doppler measurements of MPVPs based on the manufacturer and sized at this stage are summarized in Table 3.

**Table 2:** Normal parameters of mechanical prosthesis within follow-up points

Parameters	First Follow-up (1 week)	Second Follow-up (4 week)	*P*-value
	*N* = 108	*N* = 69	
Peak Pressure Gradient	18.51 (17.14–19.88)	19.07 (16.49–22.01)	0.717
Mean Pressure Gradient	10.88 (9.85–11.97)	11.52 (9.89–13.41)	0. 526
Peak Velocity	1.97 (1.89–2.05)	2.02 (1.89–2.14)	0.629
Pulmonary velocity acceleration time	87.35 (84.49–89.97)	95.97 (89.82–102.37)	0.038
Doppler velocity index	0.67 (0.57–0.64)	0.56 (0.52–0.61)	0.229
Effective Orifice Area	2.98 (2.80–3.16)	2.79 (2.57–2.98)	0.397
Effective Orifice Area /Body surface area	1.81 (1.68–1.91)	1.72 (1.56–1.86)	0.559

Data represented as mean and 95%confidence interval

**Table 3:** Values of hemodynamic parameters in normal functioning mechanical prothesis based on valve type and sizes within first follow-up

**Valve Subtypes**	**Number**	**Size** **(mm**)	**PPG** **(mm Hg)**	**MPG** **(mm Hg)**	**PV** **(m/sec)**	**PVAT (ms)**	**DVI**	**EOA (cm**^**2**^**)**	**EOA/BSA (cm**^**2**^**/m**^**2**^**)**
St. Jude	2	21	20 (19–21)	12 (11–13)	2.15 (2.10–2.20)	97.5 (85–110)	0.50 (0.50–0.50)	1.73 (1.73–1.73)	1 (0.98–1.02)
	18	23	20 (7–35)	11 (3.5–20)	2 (1.20–2.90)	87 (52–118)	0.60 (0.24–0.90)	2.49 (1–3.74)	1.64 (0.77–2.60)
	43	25^a^	18.09(±7.45)	10.95(±5.85)	1.96(±0.43)	82.95(±10.83)	0.59(±0.20)	2.89(±0.98)	1.78(±0.64)
	9	27	15 (6–30)	6 (3–18)	1.80 (1.20–2.70)	90 (72–116)	0.73 (0.55–0.84)	4.18 (3.15–4.81)	2.32 (1.82–3.16)
	0	29	–	–	–	–	–	–	–
	1	31	14	7	1.60	93	0.76	5.73	3.19
Carbomedics	0	21	–	–	–	–	–	–	–
	2	23	18.5 (17–20)	12 (12–12)	2.00 (1.80–2.20)	77.5 (70–85)	0.40 (0.30–0.50)	1.66 (1.25–2.08)	0.98 (0.7–2.27)
	14	25	19.50 (11–30)	11 (5–33)	2.05 (1.60–2.70)	88.5 (64–108)	0.67 (0.30–0.90)	3.26 (1.47–4.42)	1.84 (0.84–2.94)
	2	27	19 (10–28)	10 (6–14)	1.90 (1.40–2.40)	77.5 (75–80)	0.71 (0.70–0.72)	4.06 (4.01–4.12)	2.26 (2.23–2.29)
	1	29	14	7	1.70	76	0.4	2.64	1.89
	0	31	–	–	–	–	–	–	–
On-X	0	21	–	–	–	–	–	–	–
	3	23	20 (7–36)	12 (4–22)	2.20 (1.10–2.70)	112 (106–118)	0.80 (0.70–0.90)	3.32 (2.91–3.74)	2.37 (1.94–2.87)
	10	25	16.50 (7–24)	10 (3–13)	1.80 (1.30–2.40)	100 (55–118)	0.55 (0.30–0.80)	2.70 (1.47–3.93)	1.46 (0.87–2.18)
	1	27	23	13	2.20	113	0.7	4.01	1.91
	2	29	20 (18–22)	11.50 (11–12)	2.10 (1.90–2.30)	102.5 (95–110)	0.55 (0.50–0.60)	3.63 (3.30–3.96)	2.02 (1.83–2.20)
						–	–	–	–

The parameters demons trated in median and range

*PPG* peak pressure gradient, *MPG* mean pressure gradient, *PV* pulmonary velocity, *PVAT* pulmonary velocity acceleration time, *DVI* doppler velocity index, *EOA* effective orifice area, *EOA/BSA* effective orifice area division to body surface area

^a^ Data represented as mean (±SD)

In the second follow-up the hemodynamic profile was measured in 69 participants. Table 4 summarizes the findings at this time-point according to the manufacturer and sizes of the MPVPs. No patient showed signs of valvular malfunction or other events regarding their recent valve replacement. At this stage the size of right ventricle was significantly decreased to 3.81 (±0.71) (*P* = 0.006). Among the different measured hemodynamic parameters, non had significant change during the 3-week interval except PVAT which showed a significant increase(*P* = 0.038). At this time, RV showed significant reduction in size (P = 0.006) and improved function (*P* = 0.001) since the previous follow up. Moreover, no significant differences were observed regarding right ventricular systolic pressure and tricuspid regurgitation gradient. The former altered from 35.38 (±14.27) to 37.67 (±14.11), while the latter changed from 30.79 (±13.50) to 32.31 (±13.81), (both *P* > 0.05).

**Table 4:** Values of hemodynamic parameters in normal functioning mechanical prothesis based on valve type and sizes within second follow-up

**ValveSubtypes**	**Size(mm)**	**Number**	**PPG (mm Hg)**	**MPG (mm Hg)**	**PV (m/sec)**	**PVAT (ms)**	**DVI**	**EOA (cm**^**2**^**)**	**EOA/BSA (cm**^**2**^**/m**^**2**^**)**
St. Jude	21	1	21	13	2.30	85	0.6	2.08	1.22
	23	14	23.5 (10–45)	14 (6–30)	2.25 (1.50–3.20)	99.5 (65–140)	0.6 (0.3–0.9)	2.7 (1.25–3.74)	1.7 (0.73–2.91)
	25	29	13 (7–73)	8 (2.5–45)	1.70 (1.10–4.10)	82 (35–185)	0.5 (0.3–0.9)	2.45 (1.47–4.42)	1.51 (0.86–3.25)
	27	4	10.5 (6–16)	6 (4–9)	1.55 (1.10–1.90)	85.5 (75–110)	0.6 (0.4–0.7)	3.43 (2.29–4.01)	1.97 (1.27–2.21)
	29		.	.	.	.	.	.	.
	31	1	14	8	1.80	113	0.3	2.26	1.26
Carbomedics	21		.	.	.	.	.	.	.
	23	1	25	17	2.40	101	0.6	2.49	1.52
	25	7	20 (10–36)	16 (6–21)	2.20 (1.60–2.80)	121 (92–175)	.70 (0.40–0.80)	3.43 (1.96–3.93)	1.94 (1.03–3.97)
	27	1	19	11	2.10	98	0.6	3.43	1.91
	29	1	14	7	1.80	100	0.6	3.96	2.83
	31		.	.	.	.	.	.	.
On-X	21		.	.	.	.	.	.	.
	23	2	28 (20–36)	17 (12–22)	2.60 (2.20–3.00)	107.5 (96–119)	0.75 (0.7–0.8)	3.11 (2.91–3.32)	2.15 (2.08–2.21)
	25	7	19 (10–34)	12 (5–20)	2.10 (1.60–2.90)	85 (79–103)	0.5 (0.3–0.8)	2.94 (2.45–3.91)	1.72 (1.29–2.18)
	27	1	6	3	1.20	65	0.3	1.72	0.82
	29		.	.	.	.	.	.	.
	31		.	.	.	.	.	.	.

The parameters demonstrated in median and range

*PPG* peak pressure gradient, *MPG* mean pressure gradient, *PV* pulmonary velocity, *PVAT* pulmonary velocity acceleration time, *DVI* doppler velocity index, *EOA* effective orifice area, *EOA/BSA* effective orifice area division to body surface area Functioning Mechanical Prothesis Based on Valve Type and Sizes within First Follow-up

## Discussion

In this study, we have described the normal hemodynamic profile for mechanical pulmonary valve prosthesis in the postoperative period. To achieve this goal, we performed early- and late-stage evaluation of the prosthesis, since it had been previously established in other prostheses that the results obtained from evaluation may deviate significantly from those of the early evaluation. Additionally, it was revealed that when abnormal function of prosthesis is suspected, previous evaluation may aid by providing a basis for comparison [8, 9]. To determine proper values of various valve-related hemodynamic features and in order to distinguish the normal functioning prosthesis by this method, the evaluation of an ample number of normally functioning prostheses is required. In addition, previous attempts on echocardiographic evaluation of prostheses in other types of heart valves suggest that flow dependent measurements can be of use only in the case of severe prosthetic valve obstruction [10, 11]. Therefore, it is crucial to measure Doppler-echocardiography parameters, such as flow-independent parameters, EOA, EOA/BAS and DVI, in order to detect and include instances of mild obstruction [9].

PVR is the most frequent cardiac operation and reoperation performed on patients with congenital heart disease and the Bioprosthetic valves are currently the choice for PVR in adults and children suffering from pulmonary insufficiency [12–15]. Bioprosthetic valves tend to deteriorate more rapidly in children, resulting in inevitable repeat interventions [16–18]. Approximately 80% of bioprosthetic valves used in the pulmonary position will require reoperation as soon as 10 years after the initial surgery, regardless of age [17]. Due to their higher durability, implanting mechanical prosthesis can be a suitable alternative. The compulsory long-term use of anticoagulants has resulted in the limited application of mechanical valves. However recent attempts at PVP assessment have reported promising long-term results [19]. In contrast and from an echocardiographic approach as well as post-surgical surveillance, these prostheses have been less ventured upon.

Characterization and normal hemodynamic values of pulmonary valve prostheses is limited to pulmonary homograft valve conduits or xenograft pulmonary prosthesis [6]. Data on normally functioning MPVPs have been reported in a number of studies, albeit with limited cases [20, 21]. In a study by Novaro et al., 51 pulmonary prosthesis were evaluated out of which only 2 were mechanical, one SJ and one Björk-Shiley and both 25 mm in diameter [20]. In an analysis of 40 pulmonary prosthesis by Sadeghpour et al., only 13 were mechanical valves and three out of these mechanical valves lacked proper function due to the presence of massive obstructive thrombosis [21].

Patient-prostheses mismatch and the selection of prosthesis valve size are of vital importance and in our study, we found a positive correlation between valve size with EOA/BSA ratio. However, in contrast to the study by Sadeghpour et al. [21], which showed a significant correlation between MPG and valve sizes, a weak, marginally positive correlation was observed in our results (*P* = 0.071). The reported coefficient in our study does not represent a strong relation. This may arise from differences in types of prosthesis models and discrepancies between the actual prosthesis size and that labeled by the manufacturer. A similar positive correlation between valve size with EOA/BSA ratio was observed in other heart prosthesis [22].

The ideal time-points for postoperative transthoracic echocardiography assessment of implanted valves are at the time of discharge and 4–6 weeks after surgery [23]. This period is critical since most of the adverse effects of surgery, as well as wound and graft healing are resolved and concluded prior to the 6th week. In addition, impaired ventricular function will improve up to this timepoint [5]. Since we were unable of prolonging the study until 6 weeks after surgery, follow ups were performed at weeks 1 and 4 post-operation. Except for PVAT, measurements of hemodynamic indices did not exhibit significant alteration between the two time points and remained more or less constant. In aortic prosthetic valves, the prolonged acceleration time is a hallmark of stenosis and prosthesis malfunction [24] . In the pulmonary position, however, delayed acceleration time is associated with improved outcomes in right heart function [25]. In this position, the normal value of acceleration time in adults and children is > 100 ms > 120 ms, respectively [26]. The drastic number of lost cases may have contributed to the relatively constant values of indices obtained at the follow ups. It is important to note that this absence of change in measured values did not impact the severity of post-surgical surveillance, since the risk of thromboembolic events is high for mechanical valves, which is highest in the first 3-month after surgery [22], therefore we recommend the close observation of all patients who will undergo mechanical prosthesis placement.

### Limitations

Gradient is a flow-dependent parameter. Therefore, all factors affecting valvular flow are also capable of influencing the measured gradient. More importantly the flow is dependent to the cardiac load. The loading condition is one of such factors which may show variations in different subjects following surgery. In turn, loading condition itself is dependent on a number of other variables, such as preload and afterload and alterations in such parameters will also influence the measured gradient. The size of the prosthesis may also impact the measured gradient, although we have taken this parameter into consideration to better evaluate its impacts on the overall outcome. One major limitation regarded to doppler echocardiography is the alignment of doppler echocardiography with the prothesis. To address this, the utmost care was taken to maintain doppler echocardiography alignment with the prosthesis and reduce the θ° to the minimum amount. However, in plenty of ToF patients, the history of previous surgical events may have altered the anatomical and geometric features of the site of assessment, making it difficult, if not impossible to maintain the aforementioned alignment and hence obtaining the correct measure of gradients become considerably difficult. An important limitation of this study is was that only a select handful of valves, regarding both size and type, were available. This resulted in fewer instances of very large and very small sizes being used during the operations. Furthermore, a dramatic drop was observed in the number of cases participating for the second their second follow up, possibly impacting the results. Moreover, to establish a more sustainable profile of Doppler parameters, we recommend considering longer follow-up periods in further studies.

## Conclusion

This descriptive analysis of patients who underwent mechanical pulmonary valve replacement surgery presents previously unavailable data on the subject of hemodynamic indices of mechanical valves obtained in pulmonary position. Although the finds of this study report hemodynamic consistency throughout the study period, the high risk of coagulation after surgery mandates the recommendation of prophylactic anticoagulation therapy.

## Data Availability

The datasets used and/or analyzed during the current study are available from the corresponding author on reasonable request.

## Abbreviations

MPVP: Mechanical pulmonary valve prostheses
PVR: Pulmonary valve replacement
SJ: St. Jude
ToF: Tetralogy of Fallot
PPG: Peak pressure gradient
MPG: Mean pressure gradient
PV: Pulmonary velocity
PVAT: Pulmonary velocity acceleration time
DVI: Doppler velocity index
EOA: Effective orifice area
EOA/BSA: Effective orifice area division to body surface area
RV: Right ventricle
VTI: Velocity time integral
